# Expansão do acesso à atenção especializada: a experiência dos Consórcios Regionais de Saúde na Bahia, Brasil

**DOI:** 10.1590/0102-311XPT156925

**Published:** 2026-08-21

**Authors:** Luis Eugenio Portela Fernandes de Souza, Ana Paula Chancharulo de Moraes Pereira, Daiane Celestino Melo, Ignês Beatriz Oliveira Lopes, Nanci Nunes Sampaio Salles, Roberta Fonseca Sampaio, Luana Souza, Ana Luiza d’Ávila Viana

**Affiliations:** 1 Instituto de Saúde Coletiva, Universidade Federal da Bahia, Salvador, Brasil.; 2 Departamento de Ciências da Vida, Universidade do Estado da Bahia, Salvador, Brasil.; 3 Secretaria da Saúde do Estado da Bahia, Salvador, Brasil.; 4 Faculdade de Medicina da Universidade de São Paulo, São Paulo, Brasil.

**Keywords:** Consórcios de Saúde, Regionalização da Saúde, Atenção à Saúde, Health Consortia, Regional Health Planning, Health Care, Consorcios de Salud, Regionalización, Atención a la Salud

## Abstract

O objetivo foi analisar o processo de implantação e os efeitos dos consórcios de saúde na Bahia, Brasil, sobre o acesso à atenção especializada. Realizou-se um estudo qualitativo de múltiplos casos em três consórcios de saúde implantados em regiões distintas da Bahia. A coleta de dados ocorreu entre julho e novembro de 2022, envolveu análise documental e 51 entrevistas semiestruturadas com gestores estaduais, regionais e municipais. A análise de conteúdo foi orientada pelo modelo teórico-lógico desenvolvido para a pesquisa. A implantação dos consórcios foi viabilizada pela forte liderança do governo estadual e por incentivos financeiros, que permitiram a gestão estratégica dos conflitos políticos com os municípios. A estratégia resultou em uma significativa expansão da oferta de serviços especializados, reduzindo a dependência de contratação de serviços privados e os encaminhamentos para a capital. Contudo, persistem desafios importantes para a integração da rede, como a incipiente articulação com a atenção primária, a alta taxa de absenteísmo (30%) e a dificuldade de adequar a carteira de serviços às demandas locorregionais. A experiência baiana demonstra que a formação de consórcios é uma estratégia potente para ampliar a oferta de atenção especializada, superando barreiras políticas por meio de incentivos e negociação. No entanto, a transição de um modelo fragmentado para uma rede de atenção verdadeiramente integrada requer investimentos adicionais em mecanismos de coordenação do cuidado, de regulação assistencial e de governança que articulem os diferentes pontos de atenção em um sistema coeso e centrado no paciente. A ampliação da oferta, por si só, é uma condição necessária, mas não suficiente, para garantir a integralidade do cuidado.

## Abstract (en)

The objective was to analyze the implementation process and the effects of health consortia in Bahia, Brazil, on access to specialized care. A qualitative study of multiple cases was carried out in three health consortia implemented in different regions of Bahia. Data collection took place between July and November 2022 and involved document analysis and 51 semi-structured interviews with state, regional, and municipal managers. The content analysis was guided by the theoretical-logical model developed for the research. The implementation of the consortia was made possible by the strong leadership of the state government and by financial incentives, which allowed the strategic management of political conflicts with the municipalities. The strategy resulted in a significant expansion of the offer of specialized services, reducing dependence on contracting private services and referrals to the capital. However, important challenges persist for the integration of the network, such as the incipient articulation with primary care, the high rate of absenteeism (30%) and the difficulty of adapting the portfolio of services to local and regional demands. The Bahian experience demonstrates that the formation of consortia is a powerful strategy to expand the supply of specialized care, overcoming political barriers through incentives and negotiation. However, the transition from a fragmented model to a truly integrated care network requires additional investments in care coordination, care regulation, and governance mechanisms that articulate the different points of care in a cohesive and patient-centered system. The expansion of supply, in itself, is a necessary condition, but not sufficient, to guarantee the integrality of care.

## Abstract (es)

Este estudio tuvo como objetivo analizar el proceso de implantación de los consorcios de salud en Bahía, Brasil, y sus efectos sobre el acceso a la atención especializada. Se realizó un estudio cualitativo de casos múltiples en tres consorcios de salud implantados en diferentes regiones de Bahía. Los datos se recopilaron entre julio y noviembre de 2022 mediante análisis documental y 51 entrevistas semiestructuradas con gestores estatales, regionales y municipales. El análisis de contenido se basó en el modelo teórico-lógico desarrollado para la investigación. La implantación de los consorcios se viabilizó gracias al fuerte liderazgo del gobierno estatal y a los incentivos financieros, que permitieron la gestión estratégica de los conflictos políticos con los municipios. La estrategia resultó en una ampliación significativa de la oferta de servicios especializados, lo que redujo la dependencia de contratación de servicios privados y las derivaciones a la capital. Sin embargo, siguen existiendo importantes desafíos para la integración de la red, como la incipiente articulación con la atención primaria, la elevada tasa de absentismo (el 30%) y la dificultad de adaptar la cartera de servicios a las demandas locales y regionales. En Bahía, la experiencia demuestra que formar consorcios es una poderosa estrategia para ampliar la oferta de atención especializada, superando las barreras políticas a través de incentivos y negociación. Sin embargo, pasar de un modelo fragmentado a una red de atención verdaderamente integrada requiere inversiones adicionales en mecanismos de coordinación del cuidado, de regulación asistencial y de gobernanza que articulen los diferentes puntos de atención en un sistema cohesionado y centrado en el paciente. Ampliar la oferta es, por sí solo, una condición necesaria, pero no suficiente, para garantizar la integralidad del cuidado.

## Introdução

Desde o início de sua implantação, o Sistema Único de Saúde (SUS) vem ampliando a oferta de serviços. Com efeito, os serviços de atenção primária chegaram, em dezembro de 2023, a cobrir 79,73% da população ^1^. Essa expansão, por sua vez, aumentou a demanda por atenção especializada ^2^, o que ensejou iniciativas para expandir a oferta, articulando os serviços em redes, como a *Norma Operacional da Assistência à Saúde nº 01* ^(^ ^3^ ^)^ de 2001, o Pacto pela Saúde (2006) ^4^, a *Portaria nº 4.279* ^(^ ^5^ ^)^ de 2010 e o *Decreto nº 7.508* ^(^ ^6^ ^)^ de 2011.

No entanto, persistem as dificuldades de acesso, tanto aos serviços básicos, notadamente no que concerne à equidade, quanto aos especializados ^7^ ^,^ ^8^. Ademais, persiste o desafio de consolidar as redes regionais de saúde ^9^.

As iniquidades de acesso à atenção primária têm sido alvo de ações específicas, como o Programa Nacional de Melhoria do Acesso e da Qualidade da Atenção Básica, de 2011, e o Programa Mais Médicos para o Brasil, iniciado em 2013 e revigorado em 2023. No que tange à atenção especializada, a iniciativa mais proeminente foi o Programa Mais Acesso a Especialistas (PMAE), lançado em 2024 e incorporado ao Programa Aqui Tem Especialistas (PATE), em 2025 ^10^.

De acordo com o Ministério da Saúde, o objetivo principal do PATE é reduzir o tempo de espera por atendimento no SUS. Para isso, são adotadas oito estratégias, entre as quais: autorizar o Governo Federal a prestar atendimento especializado em apoio a estados e municípios, ampliar os turnos de atendimento na rede de saúde pública e privada e ampliar a oferta de exames, consultas e cirurgias nas unidades privadas ^10^.

Considerando a estrutura federativa do Brasil, o êxito de programas de expansão de oferta, como o PATE, exige alto grau de coordenação entre os entes federados ^11^. Para promover essa coordenação, entre outras estratégias, têm sido formados consórcios públicos. Em tese, os consórcios, voltados para o provimento de bens e serviços públicos envolvendo distintos entes federados, permitiriam a expansão da oferta, com a otimização do uso de recursos escassos ^12^.

A análise de experiências consolidadas de expansão da oferta de serviços especializados pode ser útil à definição de novas iniciativas de ampliação do acesso, como a implementação do PATE. Nesse sentido, é oportuno e relevante apresentar os resultados de uma pesquisa que analisou o processo de implantação e os efeitos dos consórcios públicos de saúde na Bahia, examinando criticamente o processo de implantação dos consórcios regionais e seus efeitos sobre o acesso aos serviços especializados e a integração da rede de atenção à saúde.

## Metodologia

### Modelização da intervenção

A análise foi orientada pela definição de um modelo teórico-lógico da intervenção, construído para delimitar o objeto de estudo. Embora o processo de implantação dos consórcios baianos não tenha se amparado em uma teoria explícita, a modelização, com base nas normas e nos discursos oficiais, foi crucial para orientar a produção de dados e a discussão dos achados.

**Figura 1:**
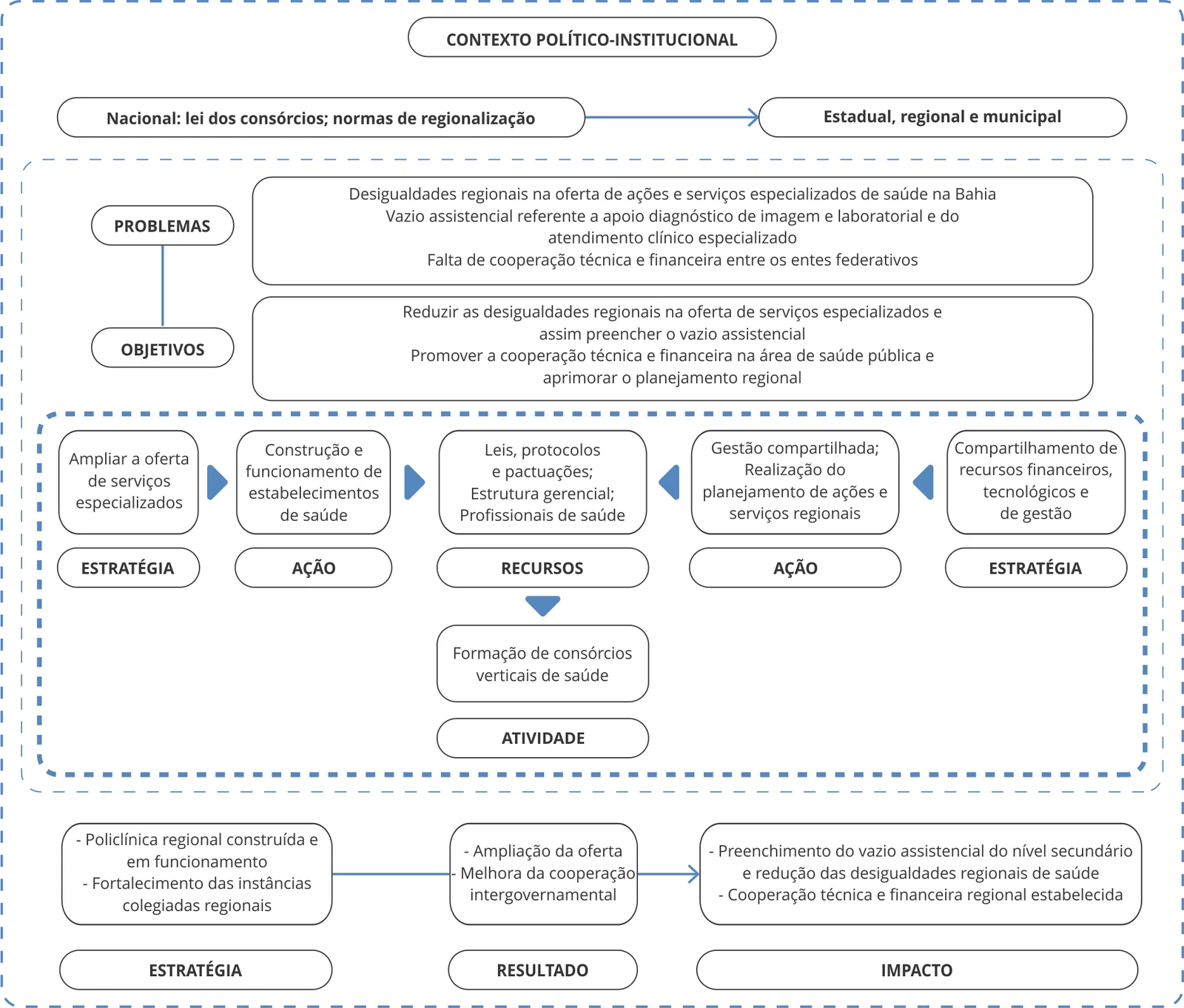
Modelo teórico da implantação dos consórcios verticais de saúde na Bahia, Brasil.

O modelo teórico da intervenção se estrutura em quatro dimensões: (1) o contexto, contemplando os aspectos legais e normativos da regionalização e a articulação federativa; (2) os problemas e objetivos relativos aos vazios assistenciais, às desigualdades regionais e à insuficiente cooperação intergovernamental; (3) a intervenção propriamente dita, ou seja, a formação dos consórcios verticais de saúde, materializada por meio de leis, acordos políticos, compartilhamento de recursos e construção de estabelecimentos de saúde; e (4) os produtos, resultados e impactos: o funcionamento da Policlínica Regional de Saúde e o fortalecimento das instâncias colegiadas regionais, a ampliação da oferta de serviços, a redução das desigualdades regionais e a consolidação da cooperação intergovernamental (Figura 1).

Para descrever os aspectos operacionais da intervenção e estabelecer as relações entre seus componentes, elaborou-se um modelo lógico da intervenção, estruturado em cinco componentes: propósitos, estrutura, processos, resultados e impacto.

**Figura 2:**
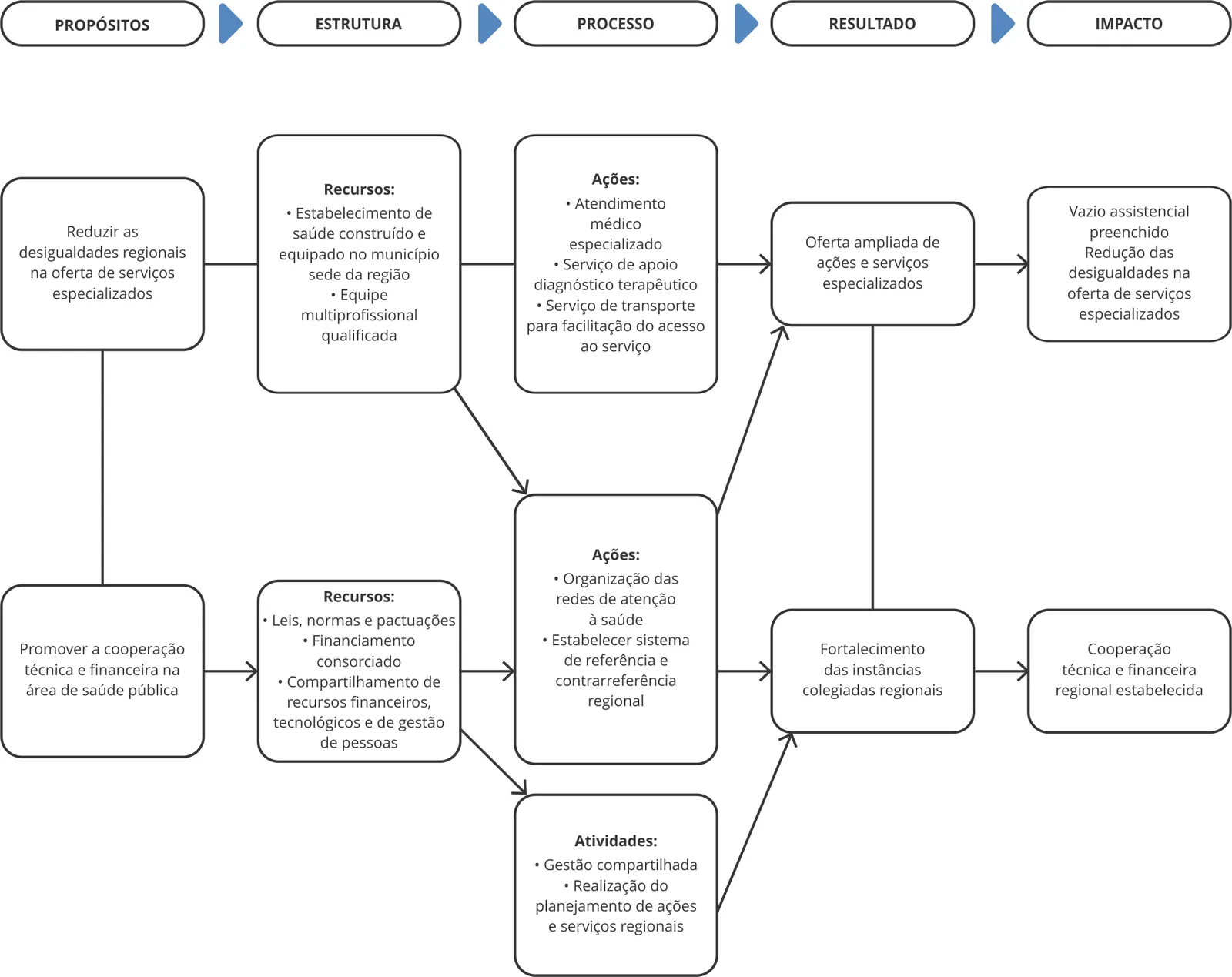
Modelo lógico da implantação dos consórcios verticais de saúde na Bahia, Brasil.

Os propósitos, coerentemente com o modelo teórico, são reduzir as desigualdades regionais na oferta de serviços especializados e promover a cooperação técnica e financeira na área de saúde pública. A estrutura detalha os elementos de suporte, como as leis, as normas, as pactuações, os recursos (financeiros, técnicos e gerenciais) e o equipamento de saúde (a policlínica). Os processos se referem ao planejamento e à gestão compartilhada, à organização da rede de serviços e do sistema de referência e contrarreferência e ao atendimento de saúde na policlínica, incluindo o transporte sanitário. Os resultados são a oferta ampliada de ações e serviços especializados e o fortalecimento das instâncias colegiadas regionais e os impactos são os vazios preenchidos, as desigualdades reduzidas e a cooperação consolidada (Figura 2).

### Desenho do estudo de múltiplos casos

Trata-se de um estudo de múltiplos casos com abordagem qualitativa, adequada para investigar fenômenos contemporâneos em seus contextos reais, especialmente quando os limites entre o fenômeno e o contexto não são evidentes ^13^. A escolha por múltiplos casos, em vez de um caso único, decorreu da opção por criar a oportunidade de identificar semelhanças e diferenças no processo de implantação e na produção de efeitos pelos consórcios em distintas regiões da Bahia, favorecendo a compreensão de fatores que influenciaram os resultados da estratégia de regionalização da atenção especializada ^14^.

Foram selecionados três casos (consórcios), com base em dois critérios: maior tempo de implantação e localização em distintas regiões de saúde da Bahia. Em cada região, o estudo incluiu o município-polo e outros dois (um de médio e um de pequeno porte, localizados a diferentes distâncias do polo), totalizando nove municípios incluídos.

A produção de dados envolveu duas técnicas: análise documental e entrevistas com informantes-chave.

O *corpus* da análise documental foi composto por documentos atinentes aos três consórcios: protocolos de intenção, estatutos, contratos de programa e de rateio, atas de assembleias, editais de licitação e editais de contratação de profissionais. Os documentos foram encontrados no sítio eletrônico do Observatório Baiano de Regionalização (https://obr.saude.ba.br/consorcios).

As entrevistas semiestruturadas abarcaram as perspectivas da gestão estadual (níveis central e regional) e da gestão municipal, totalizando 51 pessoas. Os critérios para a seleção dos informantes foram: (1) ter participado diretamente no processo de implantação dos consórcios; (2) compor a equipe gestora dos municípios, nas funções de secretário de saúde, coordenador da atenção primária à saúde (APS) e regulador; (3) exercer ou ter exercido a presidência de consórcio; ou (4) integrar a equipe diretiva da Policlínica Regional de Saúde.

Dessa forma, garantiu-se a participação de autoridades estaduais das Secretarias da Saúde, e Planejamento e da Casa Civil, de gestores regionais dos consórcios e das policlínicas, e de gestores municipais dos nove municípios. As entrevistas foram conduzidas, entre julho e novembro de 2022, em Salvador (Bahia) e em oito municípios das três regiões, a saber: Irecê, Morro do Chapéu, Lapão, Teixeira de Freitas, Vereda, Itamaraju, Jequié e Brejões. Os representantes do nono município, Ipiaú, foram entrevistados em Jequié.

Os documentos e as transcrições das entrevistas foram examinados com base na análise de conteúdo ^15^, fazendo-se a descrição sistemática do conteúdo das mensagens e a inferência de conhecimentos relativos às condições de produção e recepção dessas mensagens. A análise foi dividida em três fases. Na pré-análise, o material foi organizado em um *corpus*, com a seleção dos dados referentes à implantação e aos efeitos dos consórcios, conforme os componentes dos modelos teórico e lógico. Na fase de exploração do material, foram escolhidas as unidades de análise, a saber: as motivações, a liderança, os incentivos e os obstáculos à implantação dos consórcios (referentes ao processo de implantação) e a cobertura populacional, as desigualdades de acesso, a integração dos serviços e a regulação assistencial (referentes aos efeitos). Por fim, os dados foram tratados e interpretados, com base nas unidades de análise, e cotejados com os resultados dos estudos revisados.

O projeto de pesquisa foi aprovado pelo Comitê de Ética em Pesquisa do Instituto de Saúde Coletiva, Universidade Federal da Bahia (UFBA), em 12 de janeiro de 2021 (sob o nº 4.496.850).

## Resultados

### Breve contextualização dos casos

O consórcio de Teixeira de Freitas foi o pioneiro no estado, com o protocolo de intenção assinado em novembro de 2015. É integrado por 13 municípios, todos da mesma região de saúde. A Policlínica Regional de Saúde foi inaugurada em 17 de novembro de 2017.

O consórcio de Irecê teve seu protocolo de intenção assinado em dezembro de 2015. Sua policlínica foi a segunda a ser inaugurada, em 8 de dezembro de 2017. Dos 19 municípios da região de saúde, apenas Xique-Xique não aderiu ao consórcio. Em contrapartida, seis municípios de outras regiões decidiram integrá-lo.

O consórcio de Jequié teve seu protocolo de intenção assinado em novembro de 2015, sendo a Policlínica Regional de Saúde inaugurada em 22 de dezembro de 2017. Reunia, inicialmente, 28 municípios, mas, em 2022, o Município de Ubaitaba se desligou e passou a integrar o consórcio de Itabuna.

### O processo de implantação dos consórcios

Nesta categoria analítica, são apresentados as motivações, a liderança, os incentivos e os obstáculos à implantação dos consórcios. Por razões discutidas a seguir, os processos de implantação apresentaram características muito semelhantes nos três casos analisados, não evidenciando diferenças dignas de nota.

A análise do material empírico revelou um conjunto variado de motivos que condicionaram a adesão dos municípios à proposta de implantação dos consórcios: o aumento da oferta e a qualificação da atenção especializada com vistas a ampliar o acesso aos serviços; a melhoria da eficiência dos serviços; o aprimoramento da cooperação técnica entre estado e municípios; e o rateio de gastos entre o estado e os municípios para provisão de serviços de saúde.

“*A policlínica traz para próximo dos municípios as especialidades, o acesso a alguns exames de média complexidade*” (Referência técnica do NRS).

Um destaque reiterado foi a liderança do então governador da Bahia (2014-2022), operada por meio de articulação direta com prefeitos e deputados governistas. Essa articulação viabilizou a aprovação da *Lei nº 13.374* de 2015, que disciplina a participação do estado e dos municípios da Bahia nos consórcios verticais de saúde.

Contudo, não se tratou de um processo simples. Gestões municipais sob o comando de prefeitos filiados a partidos de oposição exigiram do Executivo estadual grande capacidade de negociação. Em 2025, dos 417 municípios do estado, apenas a capital do estado não havia aderido a um consórcio.

“[O] *idealizador, realmente, foi o Governador Rui Costa*” (Técnico da SESAB).

No que tange aos incentivos à implantação dos consórcios, a pesquisa revelou um elenco amplo, que pode ser agrupado em seis categorias: (1) o financiamento da construção e do equipamento da PRS e da aquisição de veículos pelo estado; (2) o comprometimento do estado com 40% do custeio; (3) o rateio do custeio, entre os municípios, com base no número de habitantes; (4) a perspectiva da ampliação de acesso a serviços médicos especializados; (5) a direção colegiada dos consórcios com a assembleia de prefeitos e representantes do governo estadual; e (6) a oferta de transporte para os pacientes.

Como obstáculos, os entrevistados destacaram a fragilidade dos Núcleos Regionais de Saúde (NRS), expressa pela ausência no acompanhamento sistemático das atividades dos consórcios e das policlínicas. Mencionaram também o funcionamento irregular dos conselhos consultivos, compostos pelos secretários municipais de saúde, e a desarticulação entre os consórcios e as Comissões Intergestores Regionais (CIR).

“*Desde a extinção das DIRES* [Diretorias Regionais de Saúde]*, algumas atividades foram bem enfraquecidas dentro do Núcleo* [Regional de Saúde]” (Referência técnica do NRS).

A sustentabilidade financeira das Policlínicas Regionais de Saúde foi apontada como uma dificuldade presente, resultado do crescimento contínuo de despesas. A manutenção preventiva e corretiva dos equipamentos de grande porte, em particular, onera bastante os consórcios. Consequência disso tem sido a necessidade de mais aportes financeiros do que os previstos para cobrir gastos com manutenção.

“*Tem que haver alguma forma de aumentar o recurso, porque, senão, a gente vai entrar na* (...) *falência dos consórcios*” (Presidente do consórcio).

### Efeitos dos consórcios: acesso, regulação e integração da rede

Nas três regiões estudadas, houve aumento da cobertura da assistência especializada e diminuição das desigualdades relativas ao acesso nessas regiões e na região de Salvador. De fato, o acesso ampliado levou à redução das viagens a Salvador em busca de cuidados à saúde. Contudo, o quantitativo de serviços ofertados ainda é visto como insuficiente. Há filas de espera para consultas em certas especialidades e certos exames, o que tem motivado a realização de mutirões. A falta de médicos é apontada como causa principal da insuficiência da oferta.

“*Houve uma melhora. Aumentou o acesso.* [A gente] *não manda muita gente pra Salvador, só em casos específicos do que não é contemplado aqui*” (Coordenadora da atenção básica).

A melhoria da integração dos serviços da atenção primária e da atenção secundária, com a organização do sistema de referência de pacientes, foi um efeito ressaltado pelos informantes-chave. Acrescentaram, todavia, que a integração é incipiente. Na prática, a maioria das solicitações de consultas e exames pelos profissionais da atenção primária não é acompanhada de relatório médico e, às vezes, nem mesmo de justificativa. Os resultados são encaminhamentos para PRS de demandas que poderiam ser atendidas nas unidades básicas.

“*Tem uma boa integração da atenção básica com a policlínica, mas tem situações que poderiam estar sendo resolvidas na atenção primária e não são*” (Referência técnica do NRS).

Quanto à integração da Policlínica Regional de Saúde com os hospitais regionais, o estudo evidenciou seu grau incipiente e a falta de fluxos regulares de pacientes e informações.

Os achados mostraram uma melhoria da regulação assistencial. Alguns municípios descentralizaram a regulação para as unidades básicas, e outros a mantiveram na administração central da Secretaria da Saúde. Poucos municípios contam com médico regulador. Na prática, a regulação se limita a um mecanismo de marcação de consultas e procedimentos.

Outro problema identificado se refere ao absenteísmo. Os entrevistados estimam que 30% das consultas e dos exames agendados não são realizados em decorrência do não comparecimento dos pacientes, usualmente, por falta de recursos financeiros.

“*Até 30 por cento, a gente pensa que é por falta do transporte, porque antes aqui, quando a policlínica tinha um transporte* [dentro] *do próprio município, não se faltava tanto*” (Reguladora municipal).

Além desses achados, as entrevistas permitiram identificar um efeito não previsto no modelo teórico: o crescimento dos provedores privados nos municípios-polo. Embora já houvesse uma oferta significativa de serviços privados, a instalação das policlínicas facilitou a contratação de médicos especialistas pelas clínicas privadas.

## Discussão

### Cooperação e conflito nas relações federativas

Os achados deste estudo revelam que a implantação dos consórcios na Bahia foi um processo predominantemente cooperativo. Com efeito, não há relatos de conflitos importantes. Com exceção da capital, todos os municípios do estado aderiram à estratégia, incluindo muitos dirigidos por partidos políticos de oposição ao governo estadual. Essa alta adesão, contudo, não deve ser interpretada como ausência de conflitos. Além da capital que não aderiu ao consórcio em sua região, há, ao menos, outro município baiano cuja experiência registra a ocorrência de conflito político-partidário que, todavia, não chegou a impedir a implantação do consórcio ^16^. O fato dos conflitos serem inerentes à relação federativa ^17^ ^,^ ^18^ ^,^ ^19^ não representa um impeditivo absoluto à cooperação. 

De todo modo, a alta adesão dos governos municipais a uma iniciativa do governo baiano não deve ser interpretada como ausência de conflitos, mas sim como resultado da capacidade do governo estadual de gerenciá-los estrategicamente. A liderança do então governador, operada por meio de articulação direta com prefeitos e deputados, e os grandes incentivos financeiros - como o financiamento integral da construção e do equipamento das policlínicas e o cofinanciamento de 40% do custeio - foram fatores decisivos para superar as resistências e garantir a viabilidade política do projeto. A cooperação federativa foi viabilizada não pela eliminação de conflitos, mas pela sua gestão estratégica por meio de uma combinação de incentivos e liderança política assertiva.

Assim, a análise das relações federativas revela que a formação dos consórcios pode ser uma estratégia eficaz de promoção da cooperação intergovernamental. No entanto, para além dessa arena macropolítica, a efetividade da política de regionalização depende ainda de como a nova estrutura de oferta é articulada com os processos de trabalho e a organização do cuidado no nível micropolítico. Desse modo, a discussão é deslocada agora para os desafios inerentes à integração da rede, examinando os limites e as potencialidades da estratégia.

### Da ampliação da oferta à integração da rede: avanços e desafios

Os resultados deste estudo demonstram que a implantação dos consórcios e de suas policlínicas na Bahia promoveu uma significativa expansão da oferta de serviços de atenção especializada e contribuiu para reduzir desigualdades de acesso entre a capital e o interior. A construção de 23 novas unidades de saúde com um rol de especialidades anteriormente escassas em diversas regiões do estado materializa o alcance do objetivo primário da política. Contudo, a análise à luz da literatura sobre a organização de sistemas de saúde evidencia que a ampliação da oferta é condição necessária, mas não suficiente, para garantir a integralidade e a continuidade do cuidado.

Ademais, a expansão da oferta não atende necessariamente a todas as demandas existentes, podendo gerar novas, seja pela emergência de necessidades antes latentes, seja pelo estímulo ao consumo de procedimentos. Nesse sentido, a ideia de reduzir filas apenas pela expansão da oferta de serviços é uma ilusão que deve ser, acaso presente, superada pelos gestores do SUS e pelas autoridades políticas.

O referencial teórico das Redes de Atenção à Saúde (RAS), consolidado por Mendes ^20^, postula que uma rede de saúde funcional não se resume à soma de seus elementos constitutivos, mas exige a articulação sinérgica entre eles. Para que a rede opere de forma integrada, é crucial a conexão entre os componentes da sua estrutura operacional, a saber: uma atenção primária resolutiva, uma atenção especializada e hospitalar de qualidade, sistemas de apoio diagnóstico e terapêutico eficientes, sistemas logísticos que garantam o fluxo de usuários e informações, e uma estrutura de governança que coordene o conjunto com base em uma população definida e em um modelo de atenção integral à saúde.

Os achados desta pesquisa, que apontam para desafios persistentes na regulação assistencial e na efetividade da atenção primária, sugerem que, na experiência baiana, a expansão do componente especializado não foi acompanhada pelo fortalecimento dos demais elos da rede. De fato, esses elos estão fragilizados pela insuficiente capacidade de governo das administrações municipais e pela estrutura incipiente das instâncias regionais da Secretaria Estadual da Saúde, como mencionado nas entrevistas.

A complexidade da integração do cuidado é detalhada por Khatri et al. ^21^, que a decompõem em múltiplos níveis. No nível individual, a coordenação implica a continuidade do cuidado ao longo do itinerário do paciente. No nível organizacional, requer a colaboração interprofissional e o uso de sistemas de informação integrados. Por fim, no nível de sistema, exige uma governança que promova a coordenação multissetorial. Os resultados deste estudo indicam que os consórcios baianos enfrentam desafios em todos esses níveis. A alta taxa de absenteísmo (30%), por exemplo, pode ser interpretada como uma fratura na continuidade do cuidado no nível individual, causada por barreiras de acesso. A incipiente articulação entre as equipes das policlínicas e da atenção primária, por sua vez, evidencia uma lacuna no nível organizacional. A própria definição da carteira de serviços, ainda que negociada entre os níveis central e regional, pode gerar descompassos com as necessidades locorregionais, comprometendo a equidade da atenção à saúde.

De forma mais específica, a integração entre a atenção primária e a secundária, um dos eixos críticos do manejo de condições crônicas, é um desafio global. Murtagh et al. ^22^ identificam que intervenções bem-sucedidas para promover essa integração vão muito além da disponibilização de especialistas. Incluem a formação de equipes multidisciplinares que atuam de forma conjunta, o desenvolvimento de programas de educação permanente para os profissionais de ambos os níveis de atenção e a implementação de tecnologias de *e-health* que permitam o compartilhamento de informações clínicas. A experiência baiana, ao focar na construção de novas unidades e na contratação de especialistas, parece ter avançado mais na dimensão da estrutura do que nos processos de trabalho colaborativos. A falta de uma contrarreferência sistematizada, apontada pelos entrevistados, é um sintoma claro dessa desarticulação, que impede que a atenção primária assuma a coordenação do cuidado.

Em suma, embora a estratégia dos consórcios na Bahia tenha sido bem-sucedida em expandir a oferta de serviços especializados, a análise dos achados deste estudo sugere que a transição de um modelo fragmentado para uma rede de atenção bem integrada ainda é uma meta a ser alcançada. A sustentabilidade e o impacto de longo prazo dessa política dependerão, portanto, da capacidade do estado e dos municípios de investirem não apenas em novos serviços, mas na criação de mecanismos efetivos de coordenação, regulação e governança que articulem os diferentes pontos de atenção em um sistema coeso e centrado no paciente.

## Comentários finais

A experiência dos consórcios de saúde na Bahia oferece lições importantes para futuras iniciativas de expansão da atenção especializada. O estudo demonstra que a formação de consórcios é uma estratégia potente para superar barreiras políticas na Federação brasileira, ao menos, quando acompanhada de incentivos financeiros sólidos e liderança política assertiva. No entanto, a simples expansão da oferta não garante a integração da rede nem a equidade no acesso. Para que políticas similares alcancem seu potencial máximo, é necessário investir simultaneamente em três áreas: (1) mecanismos de coordenação do cuidado que articulem as ações de promoção da saúde, de prevenção, diagnóstico e tratamento de doenças e de reabilitação da saúde nos três níveis de atenção; (2) adequação da oferta às necessidades locorregionais; e (3) governança que promova a participação das instâncias de controle social e a sustentabilidade financeira. A transição de um modelo fragmentado para uma verdadeira rede de atenção é um processo de longo prazo que requer não apenas ampliação da infraestrutura, mas também transformação nos processos de trabalho e nas relações entre os gestores, profissionais e usuários do sistema de saúde.

## Data Availability

As fontes de informação utilizadas no estudo estão indicadas no corpo do artigo.
